# Perioperative care in infective endocarditis

**DOI:** 10.1007/s12055-024-01740-7

**Published:** 2024-05-14

**Authors:** Eduard Quintana, Sara Ranchordas, Cristina Ibáñez, Polina Danchenko, Francis Edwin Smit, Carlos - Alberto Mestres

**Affiliations:** 1 Department of Cardiovascular Surgery, Hospital Clínic, University of Barcelona, Villarroel 170, 08036 Barcelona, Spain; 2 Cardiac Surgery Department, Hospital Santa Cruz, Carnaxide, Portugal; 3 Department of Anesthesiology, Hospital Clinic, University of Barcelona, Barcelona, Spain; 4 Department of Myocardial Pathology, Transplantation and Mechanical Circulatory Support, Amosov National Institute of Cardiovascular Surgery, Kiev, Ukraine; 5 Department of Cardiothoracic Surgery and The Robert WM Frater Cardiovascular Research Centre, The University of the Free State, Bloemfontein, South Africa

**Keywords:** Infective endocarditis, Diagnostic criteria, Cardiac surgery, Perioperative care, Complications

## Abstract

Patients undergoing surgery for acute infective endocarditis are among those with the highest risk. Their preoperative condition has significant impact on outcomes. There are specific issues related with the preoperative situation, intraoperative findings, and postoperative management. In this narrative review, focus is placed on the most critical aspects in the perioperative period including the management and weaning from mechanical ventilation, the management of vasoplegia, the management of the chest open, antithrombotic therapy, transfusion, coagulopathy, management of atrial fibrillation, the duration of antibiotic therapy, and pacemaker implantation.

## Introduction

The management of infective endocarditis (IE) is a matter of multidisciplinary interaction, which is currently recognized in Clinical Practice Guidelines [1]. This is a serious medical-surgical disease carrying significant morbidity and mortality despite advances in diagnosis, antibiotic therapy, surgical management, and preoperative care [2]. Current estimates indicate that 40–50% of the patients are surgical candidates partly due to the increased incidence observed worldwide in the past two decades [3–8]. It is also documented that a substantial proportion of patients might not receive an operation including, but not restricted to, poor preoperative condition, the common variable across the literature; these patients are older and have more comorbidities and, therefore, higher preoperative risk profile and a low 1-year survival [9, 10]. It is then clear that pre-, intra, and postoperative care is a fundamental part of patient management in which the experience and expertise of anesthesiologists and surgeons must be well integrated in the treating team [11].

We aimed at discussing specific relevant aspects of the perioperative management of patients requiring surgery for active IE (AIE).

## Methods

### Study design

This is a narrative review summarizing essential aspects of management during and after surgery for AIE. It is structured in a way that they are individually identified.

### Ethics

This is a literature review with no direct information retrieved from actual patients; therefore, it does not need ethical clearance from the Ethics Committee/Institutional Review Board. In this line, no written informed consent is necessary as no patients or hospital files have been individually addressed.

### Definitions

Infective endocarditis is known to be an infection of a native or prosthetic heart valve, the endocardial surface, or an indwelling cardiac device [11, 12].

Although this still is somewhat poorly defined, active IE is considered the phase of the infectious process during which the patient receives antibiotic therapy and develops a complication leading to an indication for surgical therapy [13–17].

The 2023 Duke-International Society of Cardiovascular Infectious Disease (ISCVID) criteria for diagnosis, as proposed by Fowler et al. [18], were considered. Only definite and possible cases of endocarditis were included.

There are neither clearly standardized and universally accepted definition nor protocols for early extubation after cardiac surgery. Fast-track protocols contemplate a variety of timings after surgery for extubation, usually between 3 and 12 h. In accordance with the 2016 Cochrane review, fast-track cardiac care includes “administration of low-dose opioid-based general anesthesia or use of a time-directed extubation protocol, or both,” aiming at reducing intensive care unit (ICU) and hospital length of stay [19]. The Society of Thoracic Surgeons (STS) defines prolonged ventilation after cardiac surgery as > 24 postoperative hours [20] and early extubation within 6 h [21].

### Literature search

The following terms were used to interrogate the free engine search PubMed that primarily accesses the MEDLINE database [22]: “Infective endocarditis,” “Surgery,” “Active phase,” “Perioperative care,” “Postoperative care,” “Coagulopathy,” and “Anticoagulation.” Articles were selected based on its relevance to the topic regardless of the year of publication but with focus on those published in the past 5 years.

### Additional considerations

In essence, the conduct of an operation for IE has some similarities with other high-risk surgery such as surgery for aortic dissection, which may have some impact on outcomes. This means that surgical technique has to be refined, that re-entry in the case of prosthetic valve endocarditis (PVE) has to be performed with utmost care to avoid potential injury to any mediastinal structure, etc. The surgical technique is something that needs to be adapted to the specific procedure be in the aortic, mitral, or any other valve position.

### Weaning from sedation and mechanical ventilation

Following most cardiac operations, patients arrive in the intensive care unit (ICU) anesthetized and sedated and will require mechanical ventilation according to their preoperative condition and intraoperative course. In general, the patients who are likely to require prolonged postoperative ventilation are those with preoperative congestive heart failure or requiring urgent/emergency surgery on sepsis or cardiogenic/septic shock. Adequate sedation and analgesia are essential currently and during the weaning process from the ventilator [23]. Propofol-based sedation has proved to allow earlier extubation, with consequent shorter ICU stay, in comparison with midazolam. The concomitant use of remifentanil aids in attaining adequate postoperative analgesia with hemodynamic stability [24–27]. More recently, dexmedetomidine seems to be another safe option for postoperative sedation despite its controversial impact on the length of stay [28–30]. Sedation is weaned off once standard criteria are met.

General criteria for early extubation follows the principles of general cardiac surgery with special focus on abnormal preoperative features usually present in patients with IE (such as hypoxemia due to pulmonary congestion, vasoplegia, and neurologic dysfunction). The physician in charge in the ICU or the attending surgeon makes the decision to extubate. Patients may be extubated directly from the ventilator or after a spontaneous breathing trial [23, 31, 32]. It is accepted that early extubation is associated with improved cardiac function and patient comfort, reduction in respiratory complications, ease in management, and cost savings [32] despite discussions on its influence on the length of stay [23, 33]. However, prolonged ventilation periods may be necessary due to preoperative, intraoperative, or postoperative factors, summarized in Table 1 [21, 34–36].

**Table 1:** Factors influencing on postoperative ventilation

Preoperative	Intraoperative	Postoperative
Anemia	Duration of CPB	Bleeding
Congestive heart failure	Transfusion of multiple blood products	LCOS
Emergency operation	Impaired cardiovascular performance	Sepsis
Impaired renal function		Pneumonia
COPD		Renal dysfunction
Age > 70 years		Stroke

Prolonged ventilation after cardiac surgery is common and a risk factor for mortality, especially in high-risk groups [34, 37]. Tracheostomy is indicated to improve comfort and cooperation and it is considered as it eliminates dead space, thus reducing the work of breathing which facilitates weaning from mechanical ventilation, reduces airway injury and ventilator-associated pneumonia, and allows early mobilization, speech, and oral nutrition [38–41]. Tracheostomy can be performed surgically or percutaneously. Early tracheostomy (< 14 days post operatively) seems to lead to a reduction in the duration of mechanical ventilation and ICU/hospital stay, compared to late tracheostomy. In fact, in patients with preoperative chronic debilitation and severe condition undergoing surgery, this 14-day threshold could be even lowered to facilitate early sedation removal and rehabilitation. The effect in mortality is not consistent in all studies [38, 41]. One of the main fears when performing early tracheostomy is increasing the risk of mediastinitis. The incidence of sternal wound infection across different studies is 7%, around 9% for open tracheostomy and 3% for percutaneous but with no major statistical differences according to a meta-analysis [38]. Percutaneous tracheostomy may thus be associated with a lower incidence of sternal wound infection [38, 41].

### Management of vasoplegia

Vasoplegic syndrome (VPS) is a frequent complication after cardiac surgery, especially in the case of AIE, with a reported incidence up to 44–48% and high mortality rate (30–50%) associated with multiorgan failure [42, 43]. Although there is not a consensual definition of VPS, it can be broadly described as a mean arterial pressure (MAP) < 65 mmHg, cardiac index (CI) > 2.2 l/min/m^2^, central venous pressure < 5 mmHg, left atrial pressure or pulmonary capillary wedge pressure (PCWP) < 10 mmHg, and systemic vascular resistance < 800 dyn/s/cm^5^. Refractory VPS occurs when hypotension is not corrected with vasopressor and fluid support [44, 45]. Surgical trauma and cardiopulmonary bypass (CPB) activate complex multifactorial interaction between pathways that stimulate production and systemic release of neurohumoral inflammatory mediators leading to vasodilation [44, 45]. Ischemia–reperfusion injury of heart and lungs and blood transfusion also contributes. Long CPB and aortic cross-clamp times are important risk factors for VPS [43, 46].

Sepsis is a serious condition caused by the invasion of the blood stream by toxin-producing microorganisms. Sepsis and septic shock are the major causes of morbidity and mortality in critically ill patients [47]. Patients suffering from IE often present for surgery in poor status mainly due to heart failure or persistent sepsis, particularly in staphylococcal infections [48], usually the most aggressive pathogen as any strain can induce IE [49]. A severe inflammatory reaction that involves mediators such as cytokines and inflammatory cells (polymorphonuclear neutrophils and macrophages) develops during sepsis. Clinically, a decrease in vasomotor tone and peripheral vascular resistance is detected, configurating microcirculatory disorders [50]. Some patients operated for AIE have a vasoplegic component in the presence of persistent infection documented by serial blood cultures or when they require urgent/emergency surgery due to cardiogenic/septic shock [48].

Therefore, treatment of VPS frequently begins during the operation. There are no established guidelines for the management of VPS, but current standard treatment includes fluid resuscitation and vasopressor administration, such as catecholamines with adrenergic alpha effects, mainly noradrenaline. Recently, non-catecholamine drugs like vasopressin appear to be an alternative to noradrenaline [51]. Vasopressin seems to increase systemic vascular resistance and decrease the need for cathecolamines with no additional complications [52, 53]. More recently, methylene blue, hydroxocobalamin, corticosteroids, ascorbic acid, and thiamine have been used as adjuvants. Their effect on mortality benefits is still not clear and are recommended only as rescue therapy [43, 51–55].

The role of hemoadsorption in the treatment of VPS in IE is currently controversial. Essentially, hemoadsorption is considered in surgery for AIE aiming to control the intrinsic inflammatory component with uncontrolled release of inflammatory mediators and facilitate intraoperative control. Intraoperative hemoadsorption is known to reduce plasma cytokines and the need for vasopressor support [56]. Although hemoadsorption contributes to reduce the inflammatory component and to a stable intraperative hemodyamics, its actual impact on mortality and organ dysfunction in IE is still not fully elucidated. On the one side, two randomized studies [57, 58] did not detect differences among groups as regards reduction in the Sequential Organ Failure Assessment (SOFA) score, duration of mechanical ventilation, or renal replacement therapy. Other equally recent non-randomized studies confirmed significantly reduced sepsis-associated mortality and faster recovery of hemodynamics and organ function [59, 60]. More information is needed to confirm the actual role of hemoadsorption in IE and probably a better definition of specific primary endpoints in controlled studies and appropriate patient selection seeking those who may benefit the most of such approach [57–60].

### Open chest therapy and delayed sternal closure

Leaving the chest open after a cardiac operation is a frequent decision after pediatric cardiac surgery. In the adult setting also, it aims at reducing hemodynamic and respiratory instability and assisting in the control of intraoperative uncontrollable bleeding. Compression of the heart by surrounding structures reduces diastolic filling leading to a decrease in cardiac output, more significant when poor ventricular compliance is present due to ischemia, reperfusion, and edema [61, 62]. Therefore, delayed sternal closure (DSC) is a useful tool in the management of patients that cannot tolerate chest closure. Use of DSC in adult patients has been reported as ranging from 1.7 to 5%. The main indications for DSC are hemodynamic instability, bleeding/coagulopathy, cardiac edema, and arrhythmias with hemodynamic compromise. In IE, bleeding/coagulopathy and unstable hemodynamics are the most frequent indications in our experience. Patients submitted to redo, emergency, or complex operations are more likely to require DSC [61–64].

**Fig. 1:**
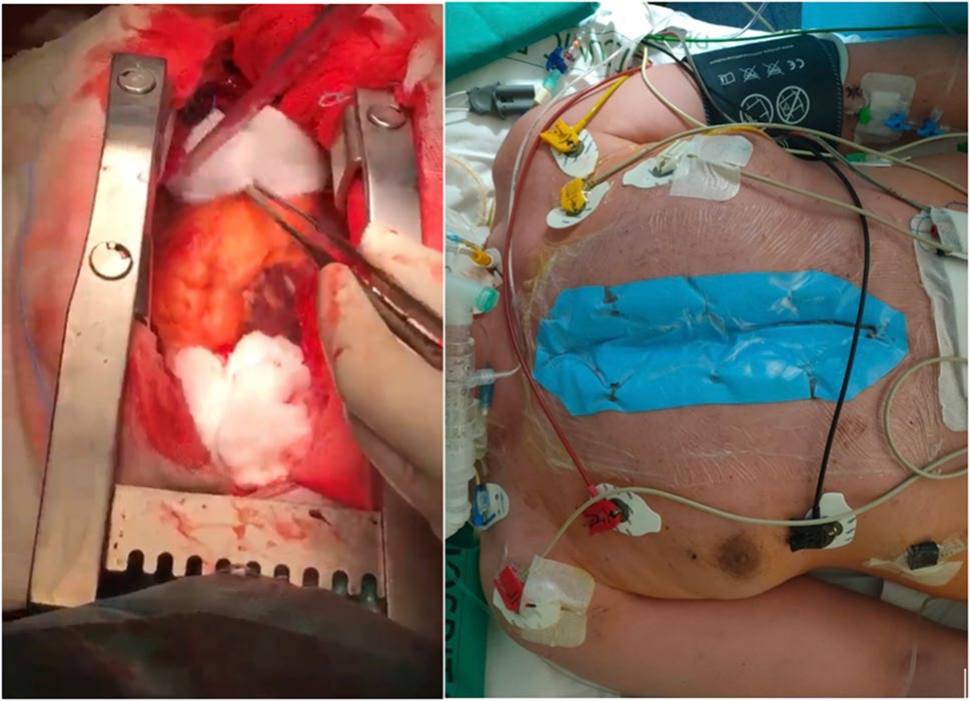
Left panel—intraoperative view of mediastinal packing with cotton, our usual practice. Right panel—postoperative view in the intensive care unit. Silicone membrane covering the defect, anchored to the skin

Various methods of DSC have been described, such as keeping the sternum open with a self-retaining retractor or a modified syringe, mediastinal packing, high-density polyethylene film coverage, silicone membrane, or sterile polytetrafluoroethylene layer sutured to the skin and primary skin closure [65] (Fig. 1). Vacuum-assisted dressing systems with a porous low-adhesive soft dressing applied on the heart for protection have also been used with minimal infectious complications [66, 67]. In the ICU, patients are kept sedated and ventilated until chest closure. Debridement and irrigation can be performed every 24 to 72 h to ensure a clean wound. This can be done in the ICU or operating room with a strict sterile technique. Usually, broad-spectrum antibiotics are maintained while the chest is open and thereafter. Used regimens vary widely among centers [61, 64–67]. Patients requiring DSC are at risk of surgical site infection because of, not only the open chest, but also the frequently prolonged CPB time, low cardiac output, excessive bleeding, and need for multiple re-explorations of the chest cavity [64]. Mortality rates are high after DSC (27–48.4%) but are mostly related to the primary indication for DSC [68].

### Antithrombotic therapy

Current strategies on pre- and postoperative anticoagulation therapies in patients with IE are controversial due to the limited number of randomized control trials (RCTs), the low incidence of this pathology, and lack of meta-analyses [69]. According to available studies, antiplatelet and/or anticoagulation management should be assessed individually by the Endocarditis Team, whose core members should include cardiologists, cardiovascular surgeons, infectious disease specialists (or internal medicine specialists with expertise in infectious diseases), and microbiologists [1, 70]. An appropriate postoperative anticoagulation strategy is crucial to improving outcomes and requires good understanding of the indication, timing, and regimen of anticoagulation in the setting of IE.

Current clinical practice guidelines on the management of IE suggest restrictive use of antiplatelet/anticoagulation agents after surgery to avoid potential hemorrhagic events [1, 70]. This strategy potentially favors patients receiving repair or biological valves at the time of surgery; however, this is less clear in the case of mechanical prostheses [1, 70, 71]. According to Pettersson et al., IE alone is not an indication to anticoagulation; nevertheless, IE patients with high thrombotic risk are more likely to benefit from anticoagulation, even if the bleeding risk is high [70]. This group of patients particularly include, among others, one of the following: mechanical heart valve; left ventricular assist device; valvular atrial fibrillation (AF) with moderate-to-severe mitral stenosis; rheumatic mitral stenosis with AF or prior embolic event or a left atrial thrombus; and non-valvular AF with CHA_2_DS_2_-VASc score ≥ 2 in men or ≥ 3 in women.

As for the antiplatelet use, the only RCT published up to date describes a salutary effect of aspirin therapy comparing oral aspirin 325 mg/day with placebo in 115 IE patients. Not only no significant benefit was observed in aspirin-treated patients regarding embolic events but an upward trend of bleeding episodes in the aspirin-treated patients [72]. Until definitive data are available, the initiation of aspirin or other antiplatelet agents as adjunctive therapy in IE is not recommended. In contrast, the continuation of long-term antiplatelet therapy at the time of development of IE with no bleeding complications may be considered [70]. Summarizing, use of antithrombotic therapy would be individualized as per the patients pre-, per-, and the postoperative factors.

### Blood transfusion thresholds

Blood transfusion requirement is frequent in AIE perioperative context. Most patients reach a non-elective intervention without the possibility of anemia optimization to increase red cell mass. Heart failure, bone marrow blockage, hospitalization, and repeat sampling are factors that cannot be timely controlled before an effective operation takes place. Although it is known that blood product transfusion is associated with impaired outcomes, this is also an effective intervention to increase blood oxygen content and viscosity and contributes to hemostasis [73]. This may be especially important during the intraoperative and early postoperative phases where the classic thresholds for red blood cell transfusion (RBC) may differ from those of more stable patients [74, 75]. There is little data to support interventions in this regard but a more liberal use of packed RBC in patients with ongoing bleeding, low-borderline cardiac output or high oxygen extraction rates may be warranted. However, allogeneic RBC transfusion is unlikely to improve oxygen transport when the hemoglobin concentration is greater than 10 g/dL and is not recommended.

Intraoperative management of patients with AIE and anemia could include hemoconcentration through CPB hemofiltration, retrograde circuit priming, routine use of cell saver, and appropriate selection of cardioplegia to minimize further hemodilution. The patients at higher risk of bleeding are usually those undergoing reoperations for PVE, because of expected complex repair such as multivalvular involvement and long CPB run time, and those with preoperative coagulopathy in the context of sepsis.

In patients with preoperative ischemic or hemorrhagic stroke, it may be reasonable to seek higher hemoglobin value. The association of anemia would support this and the higher observed rates of bleeding transformation after stroke [76]. There seems not to be a penalty associated with higher transfusion rates in patients with intracerebral hematoma [77]. However, all these assumptions and liberal strategy need to be further studied.

Once patients are stabilized, the general practice guideline recommendations on patient blood management would apply as for most cardiovascular surgery patients [78].

### Coagulopathy assessment and management

Although surgery for IE entails high risk of bleeding complications, there are no specific guidelines in this regard.

#### Preoperative assessment

One of the cornerstones for decreasing intraoperative bleeding is preoperative management of antithrombotic drugs. International guidelines recommend discontinuation of all of them for invasive procedures at high risk of bleeding. The time of last drug intake or administration to surgery depends on the type of drug. The only exception is aspirin, when prescribed in secondary prevention treatment [79].

Unfortunately, surgery for IE is often performed urgently and preoperative optimization is not possible. Nevertheless, when possible, surgery should be delayed until discontinuation interval of antithrombotic drugs is achieved. In those cases where recommended withdrawal is not possible, this information should be considered to plan the transfusion strategy. For example, anti-Xa activity might be measured in patients receiving low molecular weight heparin to determine the contribution of anti-Xa activity to bleeding. Values above 0.3 U/ml might need additional protamine. Dual antiplatelet administration effect can as well be monitored considering that there is a significant variability in the response to P2Y_12_. Thus, platelet function testing might be used to guide timing to non-emergent surgery in patients that have received P2Y_12_ inhibitors if the clinical condition allows waiting and/or to confirm the degree of platelet inhibition [79, 80].

Infective endocarditis is a clear example of the connection between the inflammatory and hemostatic systems. When bacteria enter the bloodstream, the coagulation system is activated due to bacteria recognition by receptors on endothelial cells, leucocytes, and platelets. The extrinsic coagulation pathway is activated by the release of tissue factor from endothelial cells and leukocytes. Intrinsic coagulation pathway is triggered by the activation of factor XII by bacterial wall components. The activation of both coagulation pathways leads to the formation of thrombin and thus fibrin and more platelet activation. Furthermore, there is an inhibition of the fibrinolytic and natural anticoagulation systems [81, 82]. Patients with IE have systemic hypercoagulability. However, this contrasts with the fact that one of the major complications of IE surgery is severe coagulopathy and bleeding. Several authors tried to study this contradiction in the clinical setting. Koltsova et al. [83] studied the preoperative hemostatic profile of patients with IE with standard coagulation assays, thromboelastography, thrombodynamics, and cytometry. The majority had a profile of hypercoagulability and platelet activation; nevertheless, some patients showed a hypocoagulability profile. They hypothesized that this second group entered in the phase of consumption coagulopathy. Czerwińska-Jelonkiewicz et al. [82] studied the preoperative and postoperative hemostatic profile with the point of care Total Thrombus formation Analysis System® (Fujimori Kogyo Co., Ltd., Tokyo, Japan) and found that most patients had an hypocoagulability profile with reduced hemostatic capacity before surgery that was aggravated due to prolonged activation of hemostasia, prolonged clot growth, and impaired clot stability. Breel et al. [84] compared ROTEM*®* preoperative and postoperative parameters between patients with and without IE and found that patients with IE had prolonged EXTEM clotting time but EXTEM clotting firmness parameters were increased.

These examples show that despite the general concept that IE is associated with a hypercoagulability state, patients might also have a hypocoagulability profile preoperatively, which will be aggravated after complex surgery with prolonged CPB. Therefore, we could hypothesize that preoperative profile characterization might help us to detect patients at higher risk of bleeding and plan, anticipate, and individualize our transfusion strategy.

#### Coagulation monitoring and thresholds for transfusion

Recent guidelines [79, 80] recommend the use of standard laboratory test in combination with point of care (POC) hemostatic testing, such as thromboelastography or thromboelastometry, in cardiac surgery. Moreover, they suggest developing hemostatic algorithms with predefined intervention triggers. Whether different procedures should have the same transfusion thresholds are still unsolved questions. The guidelines do not mention specific thresholds except for plasma Clauss fibrinogen level (< 1.5 g/l) and platelet transfusion to obtain a platelet count above 100.000/µL in high-risk situations such as IE.

#### Coagulopathy treatment

Prophylactic administration of antifibrinolytic therapy with tranexamic acid or e-aminocaproic acid is recommended in all cardiac surgery procedures with CPB to reduce postoperative blood loss and transfusion requirements. There are several protocols depending on the type of procedure. A high-dose protocol should be used in the context of IE. Aprotinin has been used also as an antifibrinolytic agent in cardiac surgery. This drug was withdrawn from the European market in 2008 for safety concerns but was reintroduced in 2012. Infective endocarditis is not included in its current licensing; however, it has been used off-label [79].

The products used for the treatment of bleeding in IE do not differ from other cardiac surgery procedures, namely fibrinogen concentrate or cryoprecipitate for hypofibrinogenemia. In case of coagulation factors deficiency, prothrombin complex concentrate (PCC) might be preferred over fresh frozen plasma as it is readily available. Platelet transfusion is indicated for low platelet count and/or dysfunction. Desmopressin is not systematically recommended but it might be used in bleeding patients with suspicion of platelet dysfunction [79]. Guidelines also mention two special situations: (1) in patients receiving ticagrelor or rivaroxaban, hemoadsorption may be considered; (2) in patients with refractory bleeding despite conventional hemostatic therapy, rFVIIa may be considered. The doses recommended are lower (20–40 mcg/kg) than doses used for congenital hemostatic deficits [80]. At times, clinical behavior of hemostasis of these patients in the operating theater escape from conventionally dictated management—including POC evaluations—leading to empirical replacement of coagulation factors and platelets. As in all other bleeding context, these therapies should be accompanied with maintenance of adequate hemoglobin, calcium, pH, temperature, and blood pressure.

In certain cases, we have observed persistent and uncontrollable oozing which leads to the use of external compression with sponges or cotton. We believe this allows containing sources of bleeding that may lead to increased transfusion and re-explorations in this vulnerable population. Allowing the patient to gradually correct coagulopathy may add value to postoperative management through a strategy of DSC [65].

### Repeat blood cultures and duration of antibiotics

Patients require early sequential blood culture surveillance to ensure cardiac and extracardiac (metastatic) infection control. It is reasonable to obtain a new set of antibiotics 48–72 h after surgery in patients that are evolving satisfactorily. It is exceptional that after an appropriate cardiac intervention, the source of persistent positive blood cultures remains at the valve level. In the event uncontrolled infection is documented at repeat blood cultures, whole-body computed tomography is justified to rule out the presence of other foci that may require intervention (spondylodiscitis, splenic abscess, retroperitoneal abscess, etc.). Duration of antibiotics after successful surgery for AIE is based on results from retrieved surgical specimens, which is mandatory. Appropriate therapy has positive impact on the risk of recurrence, relapse, and infection-related mortality. The three major features are a correct dose, the antimicrobial agent, and its duration. However, there are concerns as prolonged therapy may be associated to adverse events such as neutropenia, eosinophilia, rash, and *Clostridiodes difficile* infection due to disruption of microbiome [85]. Furthermore, and despite its beneficial event, it seems that prolonged therapy has no significant effect on recurrence or mortality [86]. On the other hand, short courses of postoperative antibiotic regimes did not result in differences in mortality, relapse, or reinfection in specific cases of IE [86, 87]. Despite this, the total duration of antibiotics will be counted since the start of appropriate antibiotic regime to the causative agent. However, if sample cultures obtained intraoperatively are still positive, the clock is reset at day 0 from the operation and a new antimicrobial course will be started. It is important to differentiate culture-positive scenarios from obtaining a positive result at molecular tests (e.g., 16S PCR) as genetic material may remain longer despite non-viable bacteria and this should not alter duration of postoperative antibiotics. Transition to oral antibiotics and early discharge should be considered once the patients reach stability from a medical and cardiovascular surgical standpoint. In summary, there are still no established guidelines as regards the duration of postoperative antibiotic therapy, but recent guidelines support new therapy course when the valve culture is positive [1]. Although individual institutional practices may also vary and in the absence of fever of other signs of suspected infection, blood cultures will be performed before discharge and at 3, 6, and 12 months. With regard to the expected duration of antibiotic therapy after surgery, drug treatment of PVE should last longer (≥ 6 weeks) than that of native valve endocarditis (NVE) (2–6 weeks) but is otherwise similar. Those regimens may change according to the pathogen as specific antibiotic associations might be required. In NVE needing valve replacement during antibiotic therapy, the post-operative antibiotic regimen should be that recommended for NVE, as defined by guidelines [1].

### Atrial fibrillation management

Frequently, the need for re-establishing anticoagulation in patients with history of atrial fibrillation is a challenging decision during the postoperative management of AIE. There is not existing guidance for the specific IE population, leading to arbitration with clinical judgment. This is a major issue but, in general, most of the factors that impact on the development of postoperative AF in AIE are like other type of surgery and include, but are not restricted to, older age, history of heart failure, and valve repair or replacement, with or without coronary artery bypass graft. As discussed in the literature and from own experience, a proportion of AIE patients are old and have prior operations, among other non-specific factors.

When initiating antithrombotic therapy, potential risk for bleeding requires assessment. Non-modifiable and partially modifiable bleeding risks are often specific drivers of bleeding events in patients with AIE. Certain variables associated with bleeding complications of patients with IE escape the typical risk scores applied to the general population (extensive mediastinal surgery, general inflammation, preoperative embolism, anemia, bone marrow blockage, need for medical instrumentation, etc.).

In stable patients with a low bleeding risk profile, reinstitution after surgery of anticoagulation should preferably be performed with easy-to-reverse agents (unfractioned heparin), recommended before transitioning to more definitive regimes [88]. However, there are subgroups of high-risk patients in which the decision of deferring for a few days/weeks the initiation of anticoagulation may be the best course of action. At times, avoidance of any anticoagulation could even be a choice during the acute phase of the disease. Table 2 depicts some features associated with high risk of bleeding that could justify avoidance/delay of anticoagulation for embolic prevention in patients with history of AF and who received non-mechanical valve substitutes. Atrial fibrillation is a multifaceted problem, and a variety of drugs are involved as part of the therapy. In general, the same treatment protocols apply for AF in the IE patient and, therefore, class I and III antiarrhythmics are considered. As regards oral anticoagulation, both anti-Vitamin K and novel oral anticoagulants can be prescribed but specific types of patients might require a different protocol.

**Table 2:** Features suggesting avoidance/delay of anticoagulation early after surgery for AIE (no mechanical heart valve presence)

Preoperative ischemic stroke (particularly in moderate-large cerebral mass involvement)
Preoperative hemorrhagic stroke
Persistent anemia
Low platelet count
Pure atrial fibrillation indication in patients with occluded left atrial appendage
History of atrial fibrillation but maintenance of postoperative sinus rhythm
Frailty
Postoperative bleeding complications

Surgical occlusion or exclusion of the left atrial appendage (LAA) may be considered for stroke prevention in patients with AF undergoing surgery for AIE. This may simplify greatly postoperative discussions and management [89]. Giant left atrium, which is also a known cause of AF, is very seldom seen in patients requiring surgery for IE, unless a patient has a history of long-standing rheumatic heart disease. Other than the then mandatory LAA occlusion, the treatment of giant left atrium is also debatable but not critical in this setting.

### Timing for pacemaker implantation after surgery for endocarditis

Complete heart block is frequent in surgery for AIE and especially in surgery for PVE (7–20%). Acute IE is an independent risk factor for postoperative pacemaker implant [90, 91]. It is important to ensure appropriate capture and functioning of temporary pacing systems. Daily check of capture thresholds is recommended and may alter decisions to proceed with definitive endocavitary pacing implantation. Ideally, a definitive transvenous system should not be implanted until confirmation of negative blood cultures and preferably when major central venous lines have been already removed. The mode of pacing will depend on the preoperative rhythm of the patient. If the patient has a clear indication for immediate pacing, there are different policies, with some implanting epicardial pacemakers with ventricular and/or atrial leads. It may be helpful to leave two additional sets of epicardial electrodes in patients with complete atrioventricular block and a predicted complicated postoperative course in which definite implant may not occur in the first postoperative week.

The appropriate timing for implantation is still controversial. Some studies recommend an early implant, namely < 5 postoperative days [91]. Clinical practice guidelines recommend immediate epicardial implantation in patients with preoperative conduction abnormalities, staphylococcal infection, aortic root abscess, tricuspid involvement, or previous valvular surgery [1]. It is likely that the postoperative observation period for the decision of implanting a pacemaker might be shorter than that of 14 days advocated by others [92] as a large proportion of AIE patients will have a compelling indication for implantation. The relevance of this belongs to an appropriate clinical judgment [93] and to Endocarditis Team discussions [1, 70].

### Limitations

As this is a narrative non-systematic review, some evidence might have been missed as regards specific aspects herein discussed. As such, this contribution does not aim at establishing specific universal rules of perioperative care management.

## Conclusions

Infective endocarditis is a serious disease associated with an increased surgical risk. Patients are usually older, and their preoperative condition is poor, with impact on outcomes. Perioperative management is more complex than that in other non-complicated cardiac surgery and starts in the operating room as vasoplegic syndrome; coagulopathy and open chest therapy are to be managed frequently. Adjustments in antibiotic therapy will depend on surgical specimens and the need for postoperative permanent pacing is also increased as a function of tissue destruction and specific extended reconstructions.

## Data Availability

Data in this contribution are public. As a review article, data have been extracted from the available literature on this topic.
